# Using ZnCo_2_O_4_ nanoparticles as the hole transport layer to improve long term stability of perovskite solar cells

**DOI:** 10.1038/s41598-022-06764-w

**Published:** 2022-02-21

**Authors:** Bo-Rong Jheng, Pei-Ting Chiu, Sheng-Hsiung Yang, Yung-Liang Tong

**Affiliations:** 1 Institute of Lighting and Energy Photonics, College of Photonics, National Yang Ming Chiao Tung University, No.301, Section 2, Gaofa 3rd Road, Guiren District, Tainan, 71150 Taiwan ROC; 2 Green Energy and Environment Research Laboratories, Industrial Technology Research Institute, No.360, Gaofa 2nd Road, Guiren District, Tainan, 71150 Taiwan ROC

**Keywords:** Materials for devices, Materials for energy and catalysis, Nanoscale materials

## Abstract

Inorganic metal oxides with the merits of high carrier transport capability, low cost and superior chemical stability have largely served as the hole transport layer (HTL) in perovskite solar cells (PSCs) in recent years. Among them, ternary metal oxides have gradually attracted attention because of the wide tenability of the two inequivalent cations in the lattice sites that offer interesting physicochemical properties. In this work, ZnCo_2_O_4_ nanoparticles (NPs) were prepared by a chemical precipitation method and served as the HTL in inverted PSCs. The device based on the ZnCo_2_O_4_ NPs HTL showed better efficiency of 12.31% and negligible hysteresis compared with the one using PEDOT:PSS film as the HTL. Moreover, the device sustained 85% of its initial efficiency after 240 h storage under a halogen lamps matrix exposure with an illumination intensity of 1000 W/m^2^, providing a powerful strategy to design long term stable PSCs for future production.

## Introduction

Perovskite solar cells (PSCs) have attracted a great deal of attention from academic and industrial researchers because of their rapid development in power conversion efficiency (*PCE*) from 3.8% to 25.5% within a decade^1,2^. Perovskites are considered as ideal photovoltaic materials in solar cells due to their high absorption in the visible spectrum^3^, long carrier diffusion length^4^, high carrier mobility^5^, low exciton binding energy^6^, tunable bandgaps by exchanging atomic composition^7,8^, large area production and low cost owing to solution processability. In recent years, PSCs using multiple-cation lead halide as the absorbing layer dominate mainly because of their high stability and high reproducibility compared to single-cation perovskites like MAPbI_3_, FAPbI_3_, and CsPbI_3_. Saliba et al. reported a triple-cation perovskite material Cs_x_(FA_0.17_MA_0.83_)_1−x_Pb(I_0.83_Br_0.17_)_3_ as the active layer for fabricating PSCs^9^; the best device showed an optimized open-circuit voltage (*V*_OC_) of 1147 mV, a short-circuit current density (*J*_SC_) of 23.5 mA/cm^2^, a fill factor (*FF*) of 0.785, and a certified *PCE* of 21.17%. Moreover, the device showed a stabilized *PCE* which slowly dropped to 18% after 250 h under full illumination at room temperature. Bu et al. utilized a quadruple-cation perovskite material K_y_(Cs_0.05_(FA_0.85_MA_0.15_)_0.95_)_1−y_Pb(Br_0.15_I_0.85_)_3_ as the absorbing layer^10^. The optimized device achieved a high *PCE* of 20.56%, a *V*_OC_ of 1132 mV, a *J*_SC_ of 22.95 mA/cm^2^, and a *FF* of 0.79. Besides, the device exhibited stable conversion efficiency over 1000 h stored under ambient air (10 ± 5 RH%) without encapsulation. Hence, the utilization of multiple-cation perovskite material was adopted as the light absorber instead of single- or double-cation perovskites.

In recent years, inverted PSC (p-i-n) has been extensively investigated because of its simple device architecture, ease of fabrication, improved stability, and reduced hysteresis effect^11^. Besides, tandem cells with augmented efficiency can be accomplished by combing inverted PSCs with traditional solar cells such as silicon or copper indium gallium selenide solar cells^12,13^. To fabricate inverted PSCs, organic polymers such as poly(3,4-ethylenedioxythiophene):poly(styrene sulfonate) (PEDOT:PPS), poly(4,4’-bis(*N*-carbazolyl)-1,10-biphenyl) (PPN), poly (*p*-phenylene) (PPP), and polythiophene (PT) have been used as the hole transport layer (HTL)^14–16^. Among them, PEDOT:PSS is the most widely used organic HTL with good conductivity and availability in the area of PSCs. Moreover, PEDOT:PSS is dissolved in water or alcohols, i.e., less toxic and environmentally friendly than other polymers that require organic solvents like dichloromethane or chlorobenzene. The acidic and hygroscopic nature of PEDOT:PSS induces corrosion of transparent conducting oxides such as fluorine-doped tin oxide (FTO), which restricts the long term stability and commercialization of inverted PSCs^17,18^. These polymers cause challenges due to a susceptibility to environmental factors such as moisture and ultraviolet light exposure. Furthermore, the complicated synthesis and purification process of these materials make them very expensive and difficult for mass production. In contrast to organic polymers, inorganic hole transport materials have the advantages of high carrier mobility, superior stability, low cost, and facile preparation, such as vanadium oxide^19^, copper oxide^20^, nickel oxide^21^, and cobalt oxide (Co_3_O_4_)^22^. Bashir et al. utilized spinel Co_3_O_4_ NPs as the HTL for the fabrication of PSCs with a large-area of 70 cm^2^ to achieve a *PCE* of 11.06% and extensive stability up to 2500 h under standard one sun illumination. In addition to those common metal oxides, spinel ternary metal oxides prepared by solution process have been gradually investigated as promising hole conductors in optoelectronics and lithium-CO_2_ batteries due to their tunable optical and electrical properties^23–25^. Spinel ternary metal oxides with a chemical formula of AB_2_O_4_ contain two inequivalent cations in the lattice sites^26^. The tetrahedral and octahedral sites are occupied by divalent (A) and trivalent (B) cations, respectively, leading to the formation of antisite defects that is energetically favored and is the source of the *p*-type conductivity^27^. The advantages of such ternary metal oxides include wide optical gap, better energy level alignment, and superior electrical property for serving as the HTL in optoelectronic devices. Choy and co-workers firstly proposed a controllable deamination strategy to synthesize nickel cobaltite (NiCo_2_O_4_) NPs as the HTL in inverted PSCs^28^. The optimal NiCo_2_O_4_-based cell showed 18.23% efficiency with negligible hysteresis. Lee et al. demonstrated solution-processed copper cobaltite (CuCo_2_O_4_) as the HTL to fabricate high-efficiency inverted PSCs^26^. The best PSC revealed a *PCE* of 14.12% with negligible hysteresis and retained 71% of initial *PCE* after 96 h storage under a continuous yellow light irradiation. Apart from spinel NiCo_2_O_4_ and CuCo_2_O_4_, ZnCo_2_O_4_ has also been reported to possess several features of hole transport ability, wide optical bandgap, and solution processability^29,30^, which can serve as the photocathode for the applications in photoelectrochemical water splitting and lithium-ion batteries^31,32^. Despite being a good candidate for alternative HTLs, surprisingly, no study about the use of ZnCo_2_O_4_ as the HTL in PSCs has been reported so far. Therefore, for the first time, we attempted to prepare ZnCo_2_O_4_ NPs as an efficient HTL in PSCs, which may bring important contribution to long term stability and enhanced photovoltaic performance of PSCs due to its inorganic and hole transport nature.

In this research, ammonia was chosen as a soft base to prepare ZnCo_2_O_4_ NPs as the HTL instead of strong bases like sodium hydroxide. The as-prepared ZnCo_2_O_4_ NPs can be cast into uniform thin films with high optical transparency and decent electrical properties, which are comparable or even better than PEDOT:PSS film. To fabricate inverted PSCs, 6,6-phenyl-C_61_-butyric acid methyl ester (PC_61_BM) doped with tetrabutylammonium tetrafluoroborate (TBABF_4_) and polyethylenimine (PEI) were chosen as the electron transport layer (ETL). The device with the configuration of FTO/HTL/perovskite/TBABF_4_-doped PC_61_BM/PEI/Ag was fabricated and evaluated, while ZnCo_2_O_4_ NPs layer or PEDOT:PSS film were used as the HTL for comparison. Our results demonstrated the best *PCE* value up to 12.31% and nearly hysteresis-free photocurrents at different scan directions and voltage sweep rates when using ZnCo_2_O_4_ NPs layer as the HTL. Moreover, the device sustained 85% of its initial efficiency after 240 h storage under a halogen lamps matrix exposure with an illumination intensity of 1000 W/m^2^, revealing superior potential in photovoltaic application.

## Experimental section

### Materials

FTO-coated glass substrates (7 Ω/square) were purchased from Ruilong Optoelectrionics Technology Co., Ltd. from Taiwan. Cobalt(II) nitrate hexahydrate (Co(NO_3_)_2_•6H_2_O, purity 98–102%) and zinc(II) nitrate hexahydrate (Zn(NO_3_)_2_•6H_2_O, purity 99%) were purchased from Alfa Aesar. Aqueous ammonium hydroxide (NH_4_OH_(aq)_, 25–28 wt%) was bought from Sigma-Aldrich. High-purity perovskite precursors including lead iodide (PbI_2_, purity 99.999%), lead bromide (PbBr_2_, purity 99.99%), and cesium iodide (CsI, purity 99.9%) were purchased from Alfa Aesar. Methylammonium bromide (MABr, purity 98.0%) was bought from TCI. Formamidinium iodide (FAI, purity 98%) was brought from STAREK Scientific Co., Ltd. from Taiwan. PEDOT:PSS aqueous solution (Clevios P VP AI 4083) was purchased from Heraeus Precious Metals GmbH & Co. KG. PEI (molecular weight 25,000) was bought from Sigma-Aldrich. PC_61_BM (purity 99%) was purchased from Solenne B.V., Netherlands. Other chemicals and solvents were bought from Alfa Aesar, Acros or Sigma-Aldrich and used without further purification.

### Synthesis of ZnCo_2_O_4_ NPs

The ZnCo_2_O_4_ NPs were prepared by a chemical precipitation method. Co(NO_3_)_2_•6H_2_O (0.9312 g, 3.2 mmol) was dissolved in 16 mL of deionized (DI) water with stirring at room temperature, and NH_4_OH_(aq)_ (4.8 mL) was added dropwise into the above solution. After being sonicated for 10 min, 8 mL of Zn(NO_3_)_2_•6H_2_O aqueous solution (0.2 M in DI water) was subsequently added and stirred for 30 min. The mixture was heated to 150 °C to evaporate all solvent in the air and then sintered at 225 °C for 2 h. The synthesized ZnCo_2_O_4_ NPs were washed twice with DI water and dried at 60 °C for 4 h. To prepare ZnCo_2_O_4_/DI water dispersion, 50 mg of ZnCo_2_O_4_ NPs were dispersed in 2 mL of DI water under ultrasonicated treatment for 2 h. It is important to note that all above solutions were freshly prepared before the device fabrication.

### Device fabrication

The final device structure is FTO/ZnCo_2_O_4_ NPs or PEDOT:PSS/perovskite/TBABF_4_-doped PC_61_BM/PEI/Ag. FTO were partially removed from the substrate via etching with zinc powder and 2 M HCl_(aq)_ to generate the desired pattern. The patterned FTO substrates were cleaned stepwise in detergent, DI water, acetone, and isopropyl alcohol (IPA) under ultrasonication for 10 min each. Afterward, the FTO substrates were dried with a nitrogen flow and followed by ultraviolet (UV)-ozone exposure for 20 min. The prepared ZnCo_2_O_4_ dispersion in DI water was spin-coated on cleaned FTO glass substrates at 2000 rpm for 30 s, followed by drying at 200 °C for 15 min. For comparison, PEDOT:PSS film on the FTO substrate was prepared via spin coating at 7000 rpm for 40 s and then dried at 150 °C for 15 min. After transferring substrates into the nitrogen-filled glovebox, the perovskite solution was spin coated onto the ZnCo_2_O_4_ or PEDOT:PSS layers. For the perovskite Cs_0.05_FA_0.8_MA_0.15_Pb(Br_0.15_I_0.85_)_3_ solution used in this research, a mixture of CsI (17.5 mg), FAI (197 mg), MABr (23.8 mg), PbI_2_ (555.2 mg), and PbBr_2_ (78 mg) was dissolved in a mixed solvent (1 mL) consisting of *N,N*-dimethylformamide and dimethyl sulfoxide with a 4:1 volume ratio at 70 °C for 1 h with stirring, followed by filtration with 0.45 μm membrane filters before device fabrication. The perovskite solution was spin coated on the substrates with a spinning speed of 1200 rpm for 10 s and 4500 rpm for 20 s. After 5 s in the second spinning step, 300 μL of the anti-solvent ethyl acetate was dropped. The resulting perovskite films were annealed at 105 °C for 1 h. The PC_61_BM solution (20 mg/mL in chlorobenzene containing 0.04 mg of TBABF_4_) was spin coated at 3000 rpm for 30 s on top of the perovskite layer and then dried at 100 °C for 10 min. The PEI solution (0.1 wt% in IPA) was spin coated on top of the PC_61_BM layer at 5000 rpm for 30 s. Finally, Ag electrodes with a thickness of 100 nm were thermally evaporated on top of the PEI layer under a base pressure of 10^–6^ Torr. The active area of each device was defined by a shadow mask with an open area of 4.5 mm^2^.

### Characterization and measurement

The top-view and cross-section micrographs of samples were investigated with an ultrahigh-resolution ZEISS AURIGA Crossbeam scanning electron microscope (SEM). The surface morphology and roughness of ZnCo_2_O_4_ films were measured by a Bruker Innova atomic force microscope (AFM). The surface wettability of the different films was measured using a contact angle analyzer (CAM-100, Creating Nano Technologies Inc. in Taiwan). The morphology and size of ZnCo_2_O_4_ NPs were examined with a JEOL JEM-1400 transmission electron microscope (TEM). The Fourier transform infrared (FT-IR) spectra of ZnCo_2_O_4_ pellets were measured using a Thermo Scientific Nicolet iS-10 spectrometer. The ultraviolet photoelectron spectroscopy (UPS) measurement for ZnCo_2_O_4_ NPs was performed on a PHI 5000 VersaProbe III spectrometer. A He I (hν = 21.22 eV) discharge lamp was used as the excitation source. X-ray photoelectron spectroscopy (XPS) measurements were conducted by the same spectrometer for elemental composition analysis of ZnCo_2_O_4_ NPs. X-ray diffraction (XRD) patterns and crystallinity of samples were obtained from a Rigaku MiniFlex II X-ray diffractometer. The steady-state photoluminescence (PL) spectra of perovskites on the FTO, PEDOT:PSS, or ZnCo_2_O_4_ were measured using a Princeton Instruments Acton 2150 spectrophotometer. A KIMMON KOHA He–Cd laser with double excitation wavelengths at 325/442 nm was utilized as the light source. The absorption and transmission spectra of samples were recorded with the same spectrophotometer using a xenon lamp (ABET Technologies LS 150) as the light source. To perform time-resolved PL (TR-PL) measurements, a 473 nm pulsed laser (Omicron) was utilized as an excitation light source. The TR-PL signals were recorded by a time-correlated single-photon counting module (PicoQuant MultiHarp 150 4 N) combined with a photomultiplier tube through an Andor Kymera 328i spectrometer. The apparatus was assembled by LiveStrong Optoelectronics Co., Ltd. from Taiwan. The current density–voltage (J-V) characteristics of the PSCs were measured under ambient environment by using a Keithley 2401 source measuring unit under AM 1.5G simulated sunlight exposure (Yamashita Denso YSS-100A equipped with a xenon short arc lamp, 1000 W) at 100 mW/cm^2^. The scan rate for J-V measurements was 20 mV/s. The external quantum efficiency (EQE) measurements were conducted using a PV Measurement QE-R instrument which was assembled by Enli Technology Co., Ltd. from Taiwan. To exploit the stability of devices, the encapsulated PSCs were constantly exposed to a halogen lamps matrix with an illumination intensity of 1000 W/m^2^ at room temperature with 40–60% relative humidity and their J-V characteristics were measured in each 24-h period.

## Results and discussion

### Characterization of ZnCo_2_O_4_ NPs

**Figure 1:**
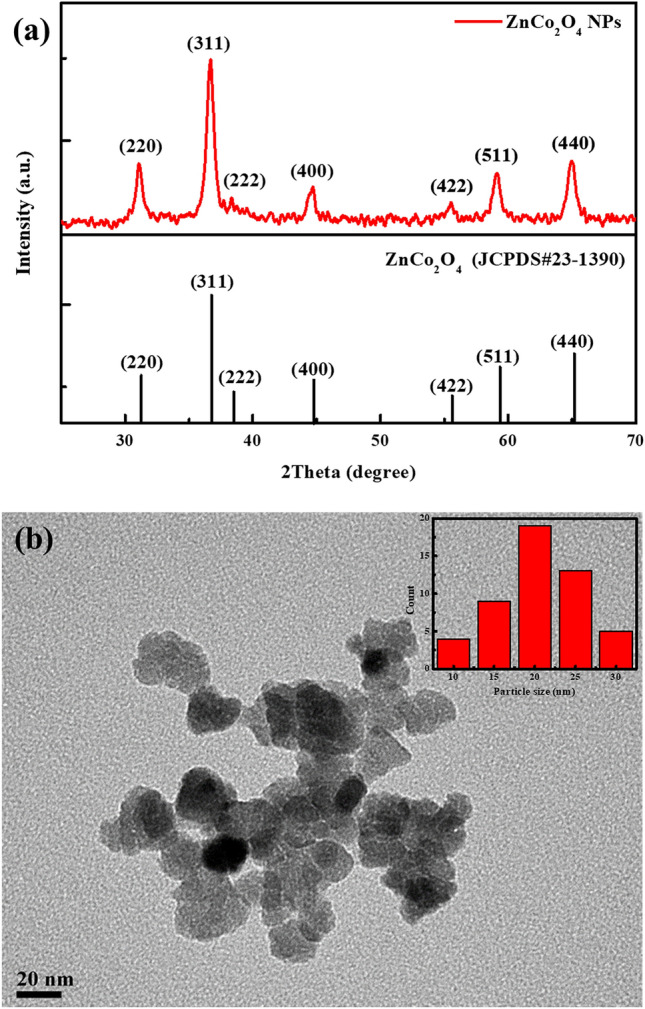
(**a**) XRD patterns and (**b**) TEM image of the ZnCo_2_O_4_ NPs. The inset shows the size distribution of NPs.

Crystallographic information of the prepared ZnCo_2_O_4_ NPs was acquired and the corresponding pattern is shown in Fig. 1a. The diffraction signals of ZnCo_2_O_4_ are found at 2*θ* = 31.06˚, 36.7˚, 38.36˚, 44.72˚, 55.52˚, 59.1˚, and 64.96˚, corresponding to the (220), (311), (222), (400), (422), (511), and (440) planes, respectively^33,34^. According to the XRD pattern, the prepared ZnCo_2_O_4_ is well consistent with the spinel phase. Figure 1b displays the TEM image of ZnCo_2_O_4_ NPs. These particles tend to aggregate with an average diameter of 20 nm.

**Figure 2:**
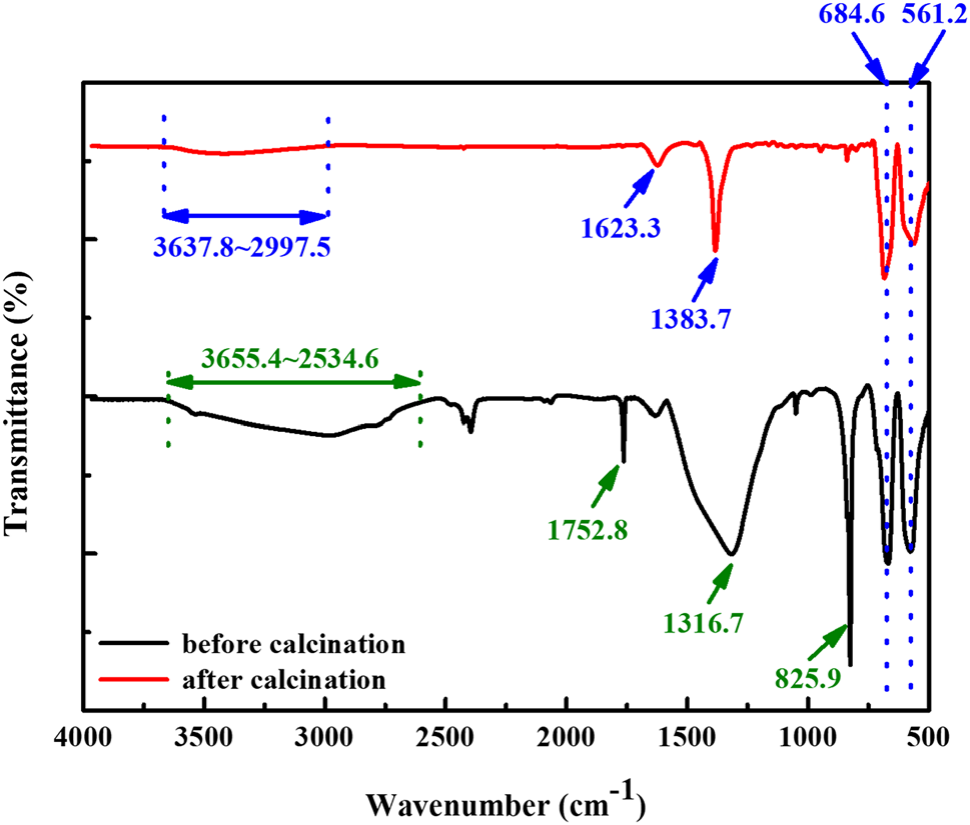
FT-IR spectra of the ZnCo_2_O_4_ NPs before and after calcination.

The residual NH_3_ molecules on the surface of ZnCo_2_O_4_ may deteriorate its electrical properties and thus should be removed. The FT-IR experiment was adopted to detect the removal of NH_3_, and the corresponding infrared spectra before and after calcination are depicted in Fig. 2. Before calcination, the characteristic stretching bands of NH_3_ molecules were observed at 3655–2597, 1753, and 826 cm^−1^, which are assigned to the N–H stretching mode, H–N–H bending vibration, and H–N–H rocking mode, respectively^35^. A significant absorption band was found at 1317 cm^-1^, which was attributed to NO_3_ groups from starting materials^36^. In addition, the two IR absorption peaks for the Zn–O and Co–O bonds were revealed at 685 and 561 cm^-1^, respectively^37^. After calcination, it is clearly seen that the absorption bands at 3655–2597, 1753, and 826 cm^-1^ were vanished, indicating that NH_3_ molecules were removed. An additional absorption band was found at 1623 cm^-1^, which was assigned to O–H bending vibration^34^. The NO_3_ absorption signal was also greatly diminished and a trace was found at 1384 cm^-1^. The Zn–O and Co–O bonds still existed at similar positions. The results proved that NH_3_ molecules can be easily removed during annealing to further improve electrical properties of ZnCo_2_O_4_ NPs.

**Figure 3:**
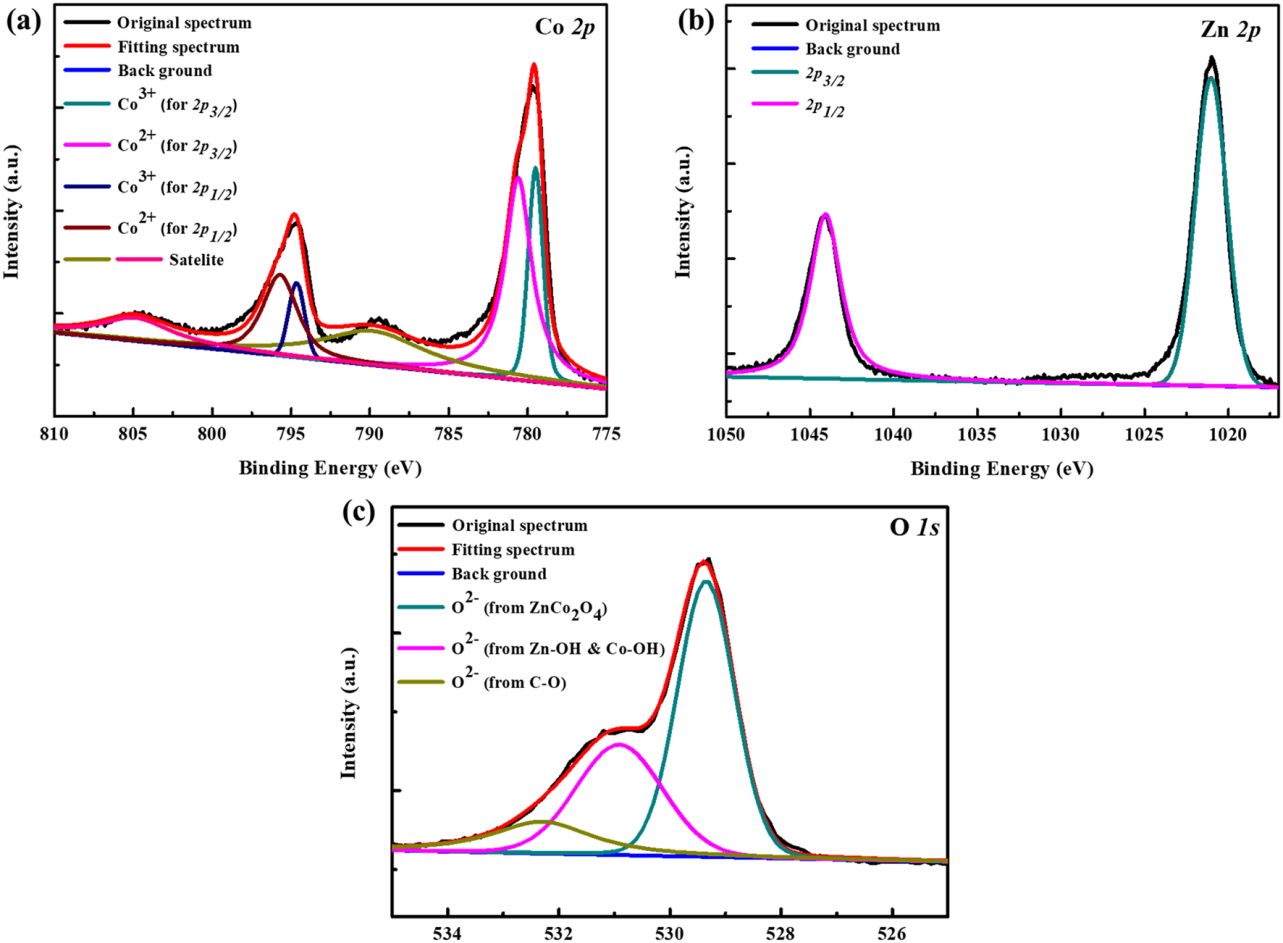
High-resolution XPS spectra of (**a**) Co *2p*, (**b**) Zn *2p*, and (**c**) O *1 s* elements in ZnCo_2_O_4_ NPs.

To identify the Zn:Co ratio in our prepared ZnCo_2_O_4_ NPs, the XPS measurements were carried out. Figure 3a shows the Co *2p* band of ZnCo_2_O_4_, and the multicomponent band can be deconvoluted into four different states at 779.5 (*2p*_3/2_), 794.7 (*2p*_1/2_) for Co^3+^, and 780.6 (*2p*_3/2_), 795.7 eV (*2p*_1/2_) for Co^2+^, and two shake-up satellite peaks at 789.8 eV near Co *2p*_3/2_ band and 804.9 eV near Co *2p*_1/2_ band. The locations of these states are in good accordance with the previous literature^38,39^. The Zn *2p* band of the spinel ZnCo_2_O_4_ NPs is depicted in Fig. 3b, revealing two XPS peaks at 1021 (*2p*_3/2_) and 1044 eV (*2p*_1/2_) for Zn^2+^ ^39^. The Zn:Co atomic ratio is calculated to be 1:2.19 based on the XPS band area, which is close to the designed ratio of ZnCo_2_O_4_ (Zn:Co = 1:2). H.Y. Chen and his coworker claimed that some Co^3+^ can occupy Co^2+^ or Zn^2+^ sites in the structure because of the similar ionic radii of Co and Zn, thus giving rise to the antisite defects (Zn_Co_)^27^, which is energetically favored for *p*-type conductivity. The prepared ZnCo_2_O_4_ NPs in this study is expected to show similar feature that is beneficial for carrier transport in optoelectronic devices. The O *1 s* spectrum of the obtained spinel ZnCo_2_O_4_ NPs is shown in Fig. 3c. The main signal due to lattice oxygen (O^2–^) is observed at 529.4 eV that is in agreement with the previous report^40^. Besides, shoulder signals at a higher binding energy of 530.9 eV and 532.3 eV come from surface hydroxyl groups and chemisorbed oxygen^41^.

**Figure 4:**
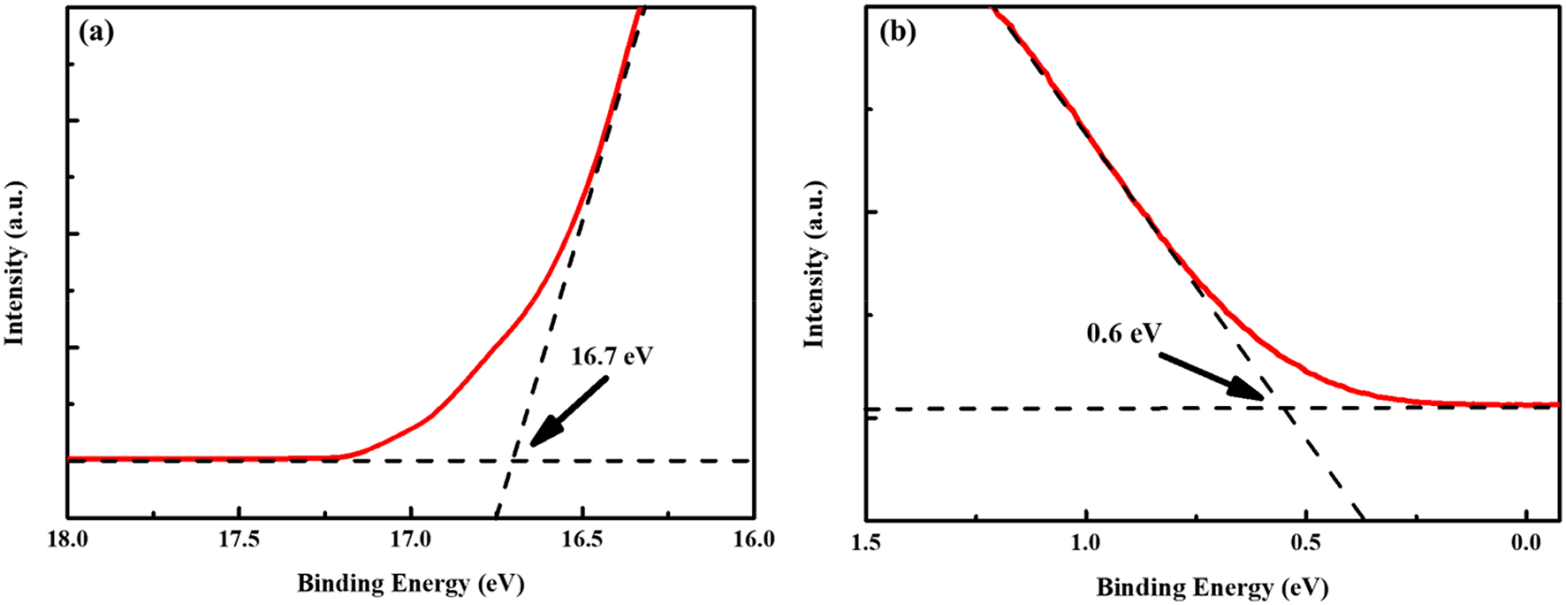
UPS spectra of ZnCo_2_O_4_ NPs at (**a**) high and (**b**) low binding energy regions. The E_F_ is obtained as E_F_ = − (21.22-high binding energy cutoff), and VB is calculated as VB = E_F_-low binding energy cutoff.

The energy levels of ZnCo_2_O_4_ NPs were calculated from their UPS spectra, as shown in Fig. 4. The work function (φ_w_) is derived by subtracting the binding energy cutoff in the high binding energy region (around 16.7 eV) from He I photon energy (21.22 eV). Since the φ_w_ is defined as the energy difference between the Fermi level (*E*_F_) and the vacuum level (0 eV), the *E*_F_ value of ZnCo_2_O_4_ NPs is determined to be −4.52 eV from Fig. 4a. Furthermore, the binding energy cutoff in the low binding energy region reveals the energy difference between the E_F_ and the valence band (VB) level^42^. The low binding energy cutoff of ZnCo_2_O_4_ NPs is found at around 0.6 eV in Fig. 4b, indicative of its VB level at −5.12 eV. Compared with the PEDOT:PSS film (VB level = −5.02 eV)^43^, the downshifted VB level (~ 0.1 eV) of ZnCo_2_O_4_ NPs is matched better with the perovskite absorbing layer, which can improve the hole extraction from the perovskite to ZnCo_2_O_4_ HTL.

### Morphological observation of the ZnCo_2_O_4_ and perovskite layers

**Figure 5:**
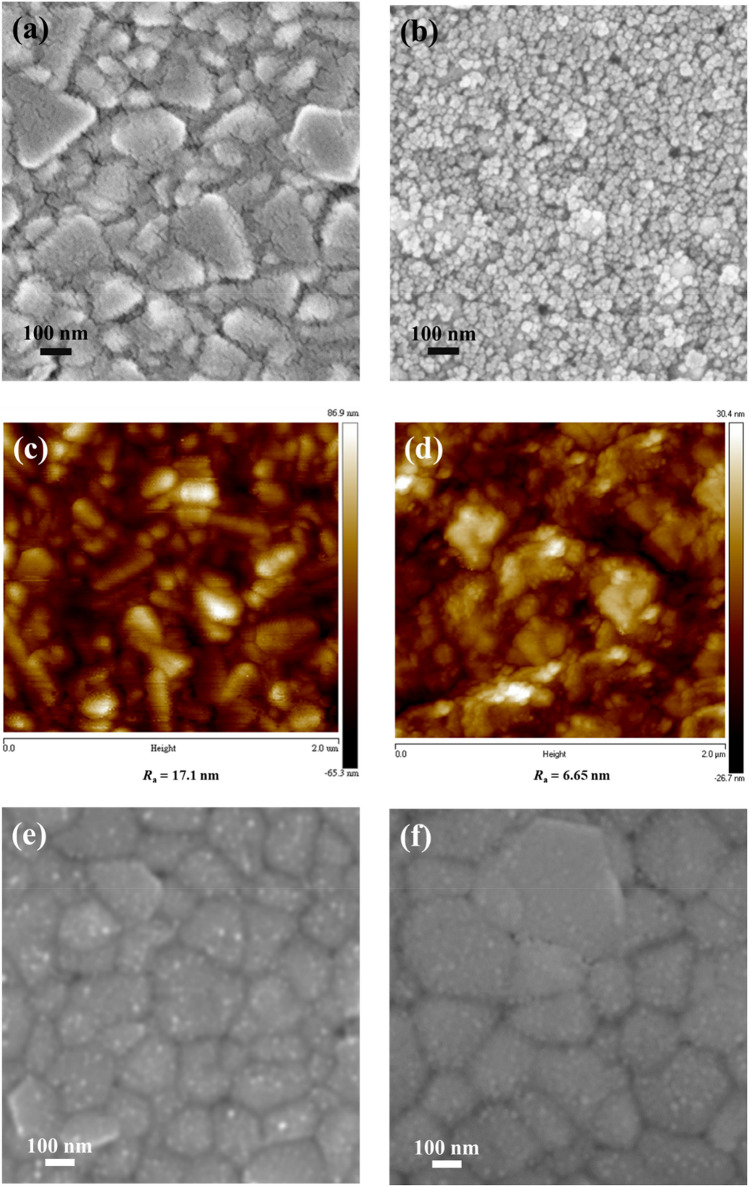
Top-view SEM and AFM topographic images of (**a**) (**c**) PEDOT:PSS film and (**b**) (**d**) ZnCo_2_O_4_ NPs layer deposited on FTO substrates. Top-view SEM images of the perovskite deposited on (**e**) PEDOT:PSS film and (**f**) ZnCo_2_O_4_ NPs layer.

The top-view SEM images of PEDOT:PSS or ZnCo_2_O_4_ NPs deposited on the FTO substrates are shown in Fig. 5a,b, respectively. A thin PEDOT:PSS layer is deposited on the top of the FTO substrate and hence the grains of low-lying FTO are clearly seen. Besides, many small cracks exist on the surface of PEDOT:PSS. In Fig. 5b, ZnCo_2_O_4_ NPs are homogeneously deposited on the FTO surface and the grains of FTO are not observable. The surface roughness of the ZnCo_2_O_4_/FTO substrate may become lower since the grains of FTO are completely covered by ZnCo_2_O_4_ NPs, as compared with the PEDOT:PSS/FTO substrate. To verify this, AFM technique was adopted to investigate the morphology and average roughness (*R*_a_) of the prepared samples. Figure 5c,d show the topographic AFM images of PEDOT:PSS and ZnCo_2_O_4_ NPs on the FTO substrates, respectively, revealing similar morphological features to those of the top-view SEM images. Furthermore, the *R*_a_ values of PEDOT:PSS or ZnCo_2_O_4_ NPs deposited on the FTO are estimated to be 17.1 and 6.65 nm, respectively. Apart from AFM investigation, contact angle experiment was also carried out to realize surface properties of the two HTLs. Figure S1a,b in the Supplementary Information represent the contact angles of a water droplet on the surfaces of PEDOT:PSS and ZnCo_2_O_4_ NPs, revealing that ZnCo_2_O_4_ NPs has a smaller contact angle of 23.6° than PEDOT:PSS film (36.8°). It is reported that the smaller contact angle facilitates the nucleation process of perovskite crystals to form a uniform layer with larger grain sizes and little pinholes^44,45^. The results from AFM and contact angle measurements reveal that ZnCo_2_O_4_ NPs can serve as a better surface modifier for FTO substrates than PEDOT:PSS, which is beneficial for improving interfacial contact and hole extraction between ZnCo_2_O_4_ NPs and the perovskite^46^. The cross-sectional SEM images of ZnCo_2_O_4_ NPs layer and PEDOT:PSS film can be seen in Figure S2a,b in the Supplementary Information, and the thickness of ZnCo_2_O_4_ NPs layer and PEDOT:PSS film was estimated to be *ca.* 65 and 40 nm, respectively.

Figure 5e and f show the top-view SEM images of the perovskite deposited on PEDOT:PSS or ZnCo_2_O_4_ NPs, respectively. No pinholes could be found for both perovskite films. The grain size of perovskite crystals on PEDOT:PSS is estimated to be in the range of 100–180 nm, while larger perovskite crystals with grain sizes of 200–300 nm were observed on ZnCo_2_O_4_ NPs, as shown in Fig. 5f. As mentioned in the previous part, the lower surface roughness of the ZnCo_2_O_4_ layer helps to form larger sizes of perovskite grains, as compared with PEDOT:PSS film^47^. The formation of larger grain size means that less grain boundary as well as reduced charge carrier recombination is obtained^48,49^. The high-quality perovskite film with fewer defects grown on ZnCo_2_O_4_ NPs is expected to exhibit higher photocurrent and conversion efficiency of PSCs, as compared to PEDOT:PSS film.

### Electrical Investigation of ZnCo_2_O_4_ NPs and PEDOT:PSS film

**Figure 6:**
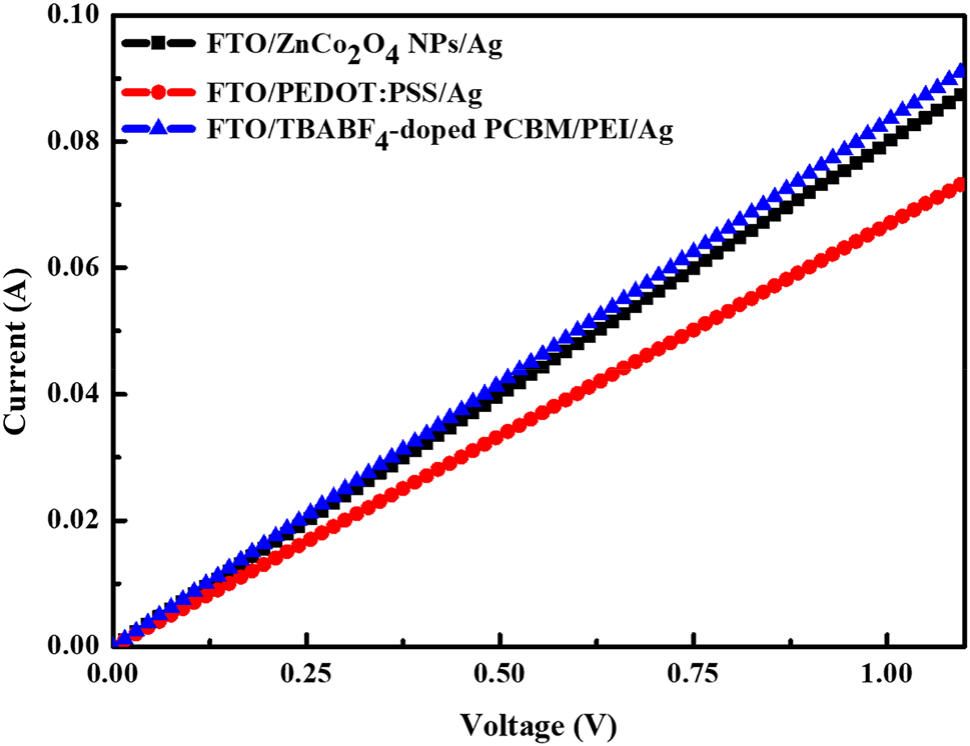
Current–voltage characteristics of hole-only devices FTO/ZnCo_2_O_4_ NPs or PEDOT:PSS/Ag and electron-only device FTO/TBABF_4_-doped PC_61_BM/PEI/Ag.

To investigate the hole transport ability of ZnCo_2_O_4_ NPs and PEDOT:PSS film, hole-only devices with the structure of FTO/ZnCo_2_O_4_ NPs or PEDOT:PSS/Ag were fabricated and evaluated. The electron-only device with the configuration of FTO/TBABF_4_-doped PC_61_BM/PEI/Ag was also fabricated for comparison. The corresponding current–voltage characteristics of the three devices are depicted in Fig. 6, indicating that the ZnCo_2_O_4_ NPs device exhibits higher current and better hole transport capability than PEDOT:PSS film. Figure S3 in the Supplementary Information displays hole mobility (*μ*_h_) of ZnCo_2_O_4_ NPs and PEDOT:PSS film, which is inferred from the space-charge limited current equation *J* = (9/8)εε_0_*μ*_h_(*V*^2^/*L*^3^). The *μ*_h_ values of ZnCo_2_O_4_ NPs layer and PEDOT:PSS film are calculated to be 9.14 × 10^–2^ and 8.52 × 10^–5^ cm^2^/Vs, respectively. The obtained *μ*_h_ of PEDOT:PSS film is close to the reported value in the literature^50^. It is seen that our ZnCo_2_O_4_ NPs layer has a hole mobility by 3 orders of magnitude higher than that of the PEDOT:PSS film. Moreover, we found that the device FTO/TBABF_4_-doped PC_61_BM/PEI/Ag shows similar current–voltage behavior to the one based on ZnCo_2_O_4_ NPs, implying equivalent carrier transport capabilities of holes and electrons in our final inverted device architecture of FTO/ZnCo_2_O_4_ NPs/perovskite/TBABF_4_-doped PC_61_BM/PEI/Ag. The balanced carrier transport also helps to reduce the hysteresis effect of devices.

### Optical investigation of ZnCo_2_O_4_ NPs and perovskite layers

Figure S4a in the Supplementary Information shows the transmission spectra of the ZnCo_2_O_4_ NPs layer and PEDOT:PSS film from 315 to 750 nm. The transmittance was measured to be 55–90% in the range of 375–650 nm and even higher over 90% in the rage of 650–750 nm for both samples with similar spectral shapes. Therefore, we speculate that the amount of incident photons entering into devices is close. The absorption spectrum of the ZnCo_2_O_4_ NPs layer is shown in Fig. S4b and its optical bandgap (E_g_) of 3.7 eV was estimated from the absorption edge around 335 nm. From UPS and absorption measurements, the conduction band (CB) level of ZnCo_2_O_4_ NPs is determined to be −1.42 eV, while the lowest-unoccupied molecular orbital (LUMO) of PEDOT:PSS is referred to the previous literature (LUMO = −3.4 eV)^51^. The relatively high CB level of ZnCo_2_O_4_ NPs can reduce electron transport from the perovskite to FTO and carrier recombination inside devices.

**Figure 7:**
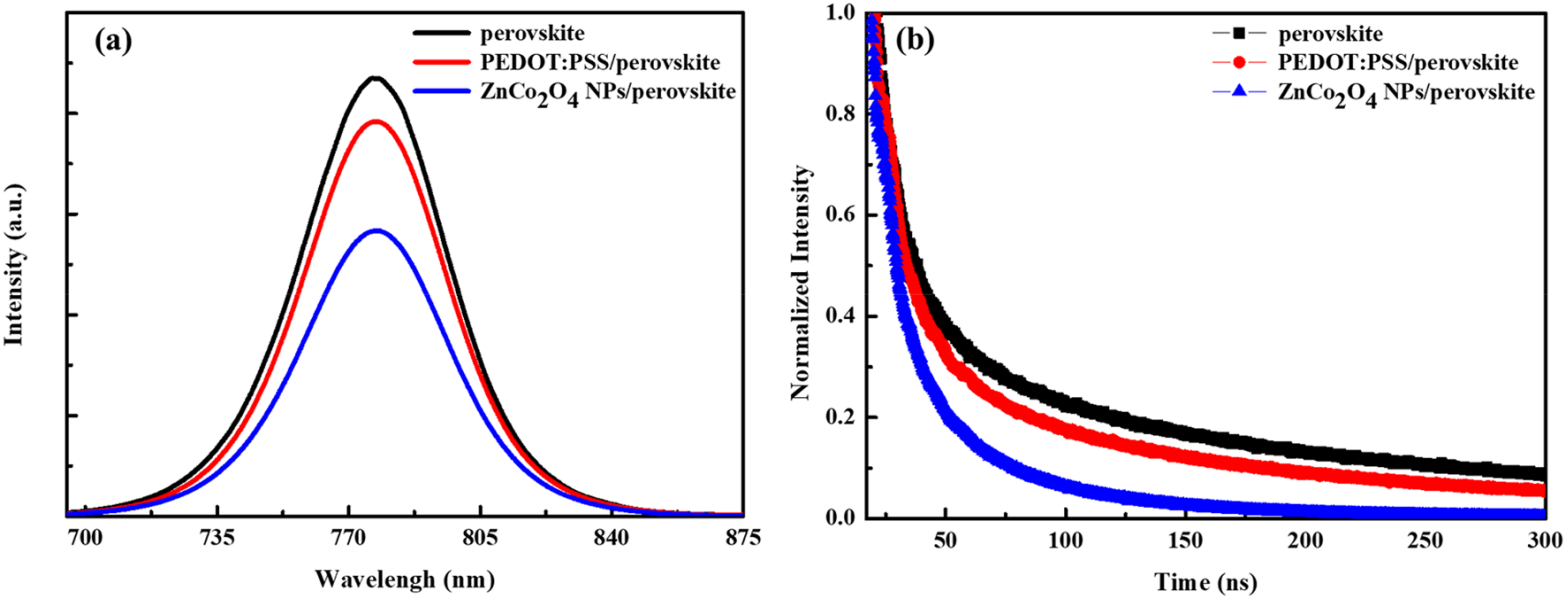
(**a**) PL emission spectra and (**b**) TR-PL decay curves of the perovskite on the FTO substrate, PEDOT:PSS film, and ZnCo_2_O_4_ NPs layer.

The steady-state PL spectra of the perovskite on the FTO substrate, PEDOT:PSS film, and ZnCo_2_O_4_ NPs layer are indicated in Fig. 7a. It is clearly seen that the perovskite deposited on the FTO substrate has the highest PL intensity, while the one on the ZnCo_2_O_4_ NPs layer owns the lowest PL emission. The reduced PL emission implies hindrance of electron − hole pair recombination and improvement of *J*_SC_ and *FF* of PSCs^26,52^. Furthermore, the TR-PL decay experiment was performed and the obtained PL decay curves of the perovskite on FTO, PEDOT:PSS film, and ZnCo_2_O_4_ NPs layer are shown in Fig. 7b. The PL decay curves agree well with a biexponential decay fitting and corresponding lifetimes of τ_1_, τ_2_, and τ_avg_ are listed in Table S1 in the Supplementary Information. It is reported that fast decay (τ_1_) originates from nonradiative capture of free carriers and the slow decay (τ_2_) comes from radiative recombination of remaining excitons^26^. The τ_avg_ is determined by the equation τ_avg_ = Σ_*i*_(A_*i*_τ_i_^2^)∕Σ_*i*_(A_*i*_τ_i_), where A_*i*_ values is derived from the fitted curve data^53^. Generally, the shorter carrier lifetime indicates more efficient charge extraction. The τ_avg_ value of the perovskite on FTO was calculated to be 107.17 ns, and it decreased to 88.61 and 39.98 ns when the perovskite was deposited on the PEDOT:PSS film and ZnCo_2_O_4_ NPs layer, respectively. This result indicates more effective charge extraction by the ZnCo_2_O_4_ NPs layer from the perovskite active layer as compared with the PEDOT:PSS film.

### Device evaluation

**Figure 8:**
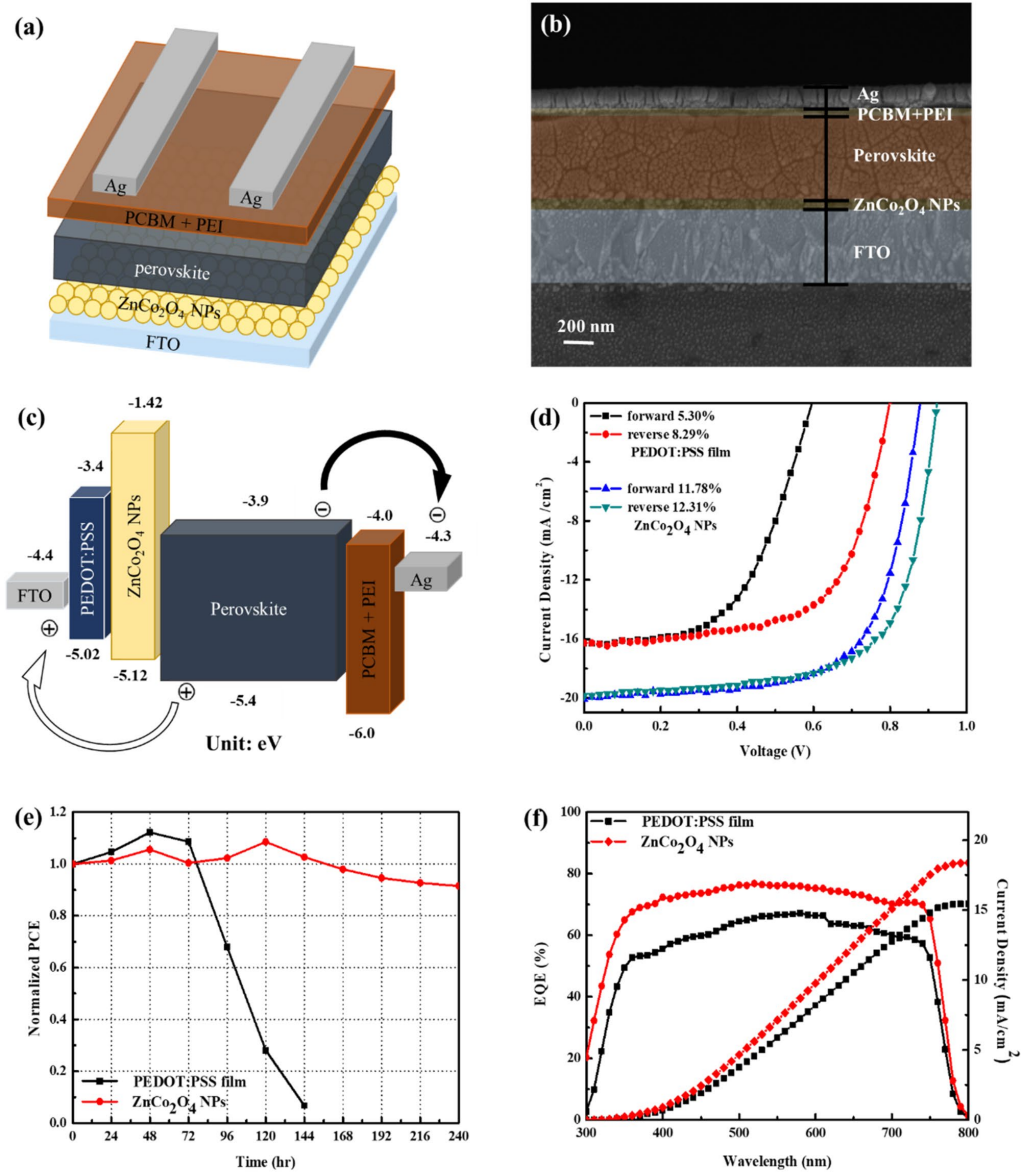
(**a**) Device structure and (**b**) cross-sectional SEM image of the PSC based on the ZnCo_2_O_4_ NPs layer; (**c**) energy level diagram of the whole device; (**d**) J-V characteristics, (**e**) normalized *PCE* evolution, and (**f**) EQE spectra and integrated current density of the PSCs based on PEDOT:PSS film or ZnCo_2_O_4_ NPs layer.

The p-i-n device structure of the inverted PSC based on ZnCo_2_O_4_ NPs HTL is shown in Fig. 8a, revealing a sandwiched architecture of FTO/ZnCo_2_O_4_ NPs/Cs_0.05_FA_0.8_MA_0.15_Pb(Br_0.15_I_0.85_)_3_/TBABF_4_-doped PC_61_BM/PEI/Ag. Figure 8b shows the cross-sectional SEM micrograph of the whole device, revealing the thickness of FTO, ZnCo_2_O_4_ NPs layer, perovskite, PC_61_BM + PEI, and Ag electrode to be 500, 60, 550, 35, and 135 nm, respectively. The energy level diagram of the whole device is illustrated in Fig. 8c. The VB and CB levels of ZnCo_2_O_4_ NPs have been discussed in the previous part, while the energy levels of the other components were referred to the previous reports^43,54,55^. In our device architecture, electrons can be successfully extracted from the perovskite absorber and transport to the Ag electrode through PC_61_BM + PEI, while holes migrate gradually from the perovskite layer through ZnCo_2_O_4_ NPs and are collected on the FTO electrode. The J-V curves of the devices measured under AM 1.5 G are shown in Fig. 8d, and the measured parameters including *J*_SC_, *V*_OC_, *FF*, *PCE*, and series resistance (*R*_S_) are summarized in Table 1. The optimized device based on ZnCo_2_O_4_ NPs showed a *V*_OC_ of 0.92 V, a *J*_SC_ of 19.85 mA/cm^2^, a *FF* of 67.19%, and a *PCE* of 12.31% in the reverse scan, which is significantly higher than the one based on PEDOT:PSS (*V*_OC_ = 0.79 V, *J*_SC_ = 17.23 mA/cm^2^, *FF* = 59.77%, and *PCE* = 8.11%). The statistical distribution of 20 individual devices for all photovoltaic parameters is depicted in Fig. S5 in the Supplementary Information. It can be seen that our devices possessed good reproducibility and PSCs based on ZnCo_2_O_4_ NPs showed relatively higher photovoltaic parameters. The improved device performance is mainly ascribed to the increased *J*_SC_ value and energy level matching between ZnCo_2_O_4_ NPs/perovskite interface. Hysteresis index (HI) can be used to describe the hysteresis behavior of PSCs according to the equation HI = (*PCE*_reverse_ – *PCE*_forward_)/*PCE*_reverse_^56^. The PSC based on ZnCo_2_O_4_ NPs has a smaller HI value of 0.043 as compared with that based on PEDOT:PSS (HI = 0.36). As a result, the reduced hysteresis of the PSC based on ZnCo_2_O_4_ NPs is in accordance with electrical measurements in the previous part. The normalized *PCE* evolution of the PSCs based on ZnCo_2_O_4_ NPs and PEDOT:PSS is shown in Fig. 8e for comparison. The PSC based on ZnCo_2_O_4_ HTL retained 85% of its initial efficiency after 240 h storage under a halogen lamps matrix exposure at room temperature, whereas the *PCE* of the device based on PEDOT:PSS HTL dropped to only 0.5% of its initial efficiency after 144 h storage. Such fast deterioration can be attributed to the acidic nature of PEDOT:PSS causing corrosion to the perovskite and FTO substrate. Therefore, the use of inorganic ZnCo_2_O_4_ HTL is highly beneficial for the device stability. As mentioned in the Introduction, the device using CuCo_2_O_4_ as the HTL retained 71% of initial *PCE* after 96 h storage under a continuous yellow light irradiation^26^. Our result reveals that ZnCo_2_O_4_ is a better candidate for the fabrication of stable PSCs. Figure 8f shows the EQE spectra and integrated current density of devices as a function of wavelength using ZnCo_2_O_4_ NPs and PEDOT:PSS as the HTL. The results demonstrate that the device based on ZnCo_2_O_4_ NPs has a higher photon-to-electron conversion capability from 300 to 750 nm compared to that based on PEDOT:PSS. The integrated current density for the devices based on ZnCo_2_O_4_ NPs and PEDOT:PSS was calculated to be 18.4 and 15.45 mA/cm^2^, respectively, which are similar to the *J*_SC_ values in Table 1.

**Table 1:** Device performance of all PSCs based on PEDOT:PSS film or ZnCo_2_O_4_ NPs as the HTL.

HTL	Scan direction	*J*_SC_ (mA/cm^2^)	*V*_OC_ (V)	*FF* (%)	best *PCE* (%)	avg *PCE*^a^ (%)	*R*_S_ (Ω)
PEDOT:PSS film	Forward	16.27	0.6	54.67	5.3	5.18	257.97
	Reverse	16.27	0.8	63.73	8.29	7.86	128.15
ZnCo_2_O_4_ NPs	Forward	20.06	0.88	66.82	11.78	11.27	109.39
	Reverse	19.85	0.92	67.19	12.31	11.55	94.51

^a^Average *PCE* values were obtained from 20 devices.

## Conclusions

In this study, we successfully synthesized ZnCo_2_O_4_ NPs by a facile chemical precipitation method, which were employed as the HTL in inverted PSCs. The obtained ZnCo_2_O_4_ NPs showed a spinel phase and an average particle size of 20 nm. The introduced NH_3_ molecules were removed by annealing process to improve electrical properties of ZnCo_2_O_4_ NPs, as verified by FT-IR experiments. The Zn:Co atomic ratio of 1:2 and *p*-type transport character were confirmed by XPS observation. The downshifted VB level of ZnCo_2_O_4_ NPs is matched better with the perovskite absorbing layer to improve the hole extraction. Smoother ZnCo_2_O_4_ NPs layer was obtained by solution process with a low surface roughness of 6.65 nm, and larger sizes of perovskite grains were formed on the ZnCo_2_O_4_ NPs layer, as compared with PEDOT:PSS film. The optimized PSC based on the ZnCo_2_O_4_ NPs HTL exhibited a high *PCE* of 12.31%, negligible hysteresis, and excellent device stability of 240 h storage under a halogen lamps matrix exposure in ambient environment. To date, the utilization of ZnCo_2_O_4_ NPs as the HTL provides a simple and effective approach to achieve PSCs with high efficiency and long term stability that show promising use in photovoltaic application.

## Supplementary Information

Supplementary Information.

## Data Availability

The datasets generated and/or analyzed in this study are available from the corresponding author upon reasonable request.

## Abbreviations

HTL: Hole transport layer
PSCs: Perovskite solar cells
NPs: Nanoparticles
*PCE*: Power conversion efficiency
*V*_OC_: Open-circuit voltage
*J*_SC_: Short-circuit current density
*FF*: Fill factor
PEDOT:PPS: poly(3,4-ethylenedioxythiophene):poly(styrene sulfonate)
PPN: Poly(4,4’-bis(*N*-carbazolyl)-1,10-biphenyl)
PPP: Poly (*p*-phenylene)
PT: Polythiophene
FTO: Fluorine-doped tin oxide
PC_61_BM: [6,6]-Phenyl-C_61_-butyric acid methyl ester
TBABF_4_: Tetrabutylammonium tetrafluoroborate
PEI: Polyethylenimine
ETL: Electron transport layer
IPA: Isopropyl alcohol
UV: Ultraviolet
SEM: Scanning electron microscope
AFM: Atomic force microscope
TEM: Transmission electron microscope
FT-IR: Fourier transform infrared
UPS: Ultraviolet photoelectron spectroscopy
XPS: X-ray photoelectron spectroscopy
XRD: X-ray diffraction
PL: Photoluminescence
TR-PL: Time-resolved PL
J-V: Current density–voltage
EQE: External quantum efficiency
φ_w_: Work function
*E*_F_: Fermi level
VB: Valence band
R_a_: Average roughness
*μ*_h_: Hole mobility
E_g_: Optical bandgap
CB: Conduction band
LUMO: Lowest-unoccupied molecular orbital
R_s_: Series resistance
HI: Hysteresis index
