# Supplementary material for: Using ZnCo2O4 nanoparticles as the hole transport layer to improve long term stability of perovskite solar cells
Source: Sci Rep. 2022 Feb 21;12:2921. doi: 10.1038/s41598-022-06764-w (PMC8861179; doi:10.1038/s41598-022-06764-w)
Supplement: Supplementary file 1 — Supplementary Information. [file 41598_2022_6764_MOESM1_ESM.docx]

**Supporting Information**

**Using ZnCo_2_O_4_ nanoparticles as the hole transport layer to improve long term stability of perovskite solar cells**

Bo-Rong Jheng,^1^ Pei-Ting Chiu,^1,2^ Sheng-Hsiung Yang,^1,^* and Yung-Liang Tong^2^

*^1^Institute of Lighting and Energy Photonics, College of Photonics, National Yang Ming Chiao Tung University, No.301, Section 2, Gaofa 3^rd^ Road, Guiren District, Tainan 71150, Taiwan R.O.C.*

*^2^Green Energy and Environment Research Laboratories, Industrial Technology Research Institute, No.360, Gaofa 2^nd^ Road, Guiren District, Tainan 71150, R.O.C.*

*Correspondence: [yangsh@mail.nctu.edu.tw](mailto:yangsh@mail.nctu.edu.tw)

**Table S1.** Lifetime parameters of TR-PL curves of the perovskite on the FTO substrate, PEDOT:PSS film, and ZnCo_2_O_4_ NPs layer.

| substrate | A_1_ (%) | τ_1_ (ns) | A_2_ (%) | τ_2_ (ns) | τ_avg_ (ns) |
| --- | --- | --- | --- | --- | --- |
| FTO | 54.61 | 13.12 | 45.39 | 119.59 | 107.17 |
| PEDOT:PSS film | 58.51 | 11.74 | 41.49 | 101.19 | 88.61 |
| ZnCo_2_O_4_ NPs layer | 54.41 | 10.15 | 45.59 | 47.58 | 39.98 |


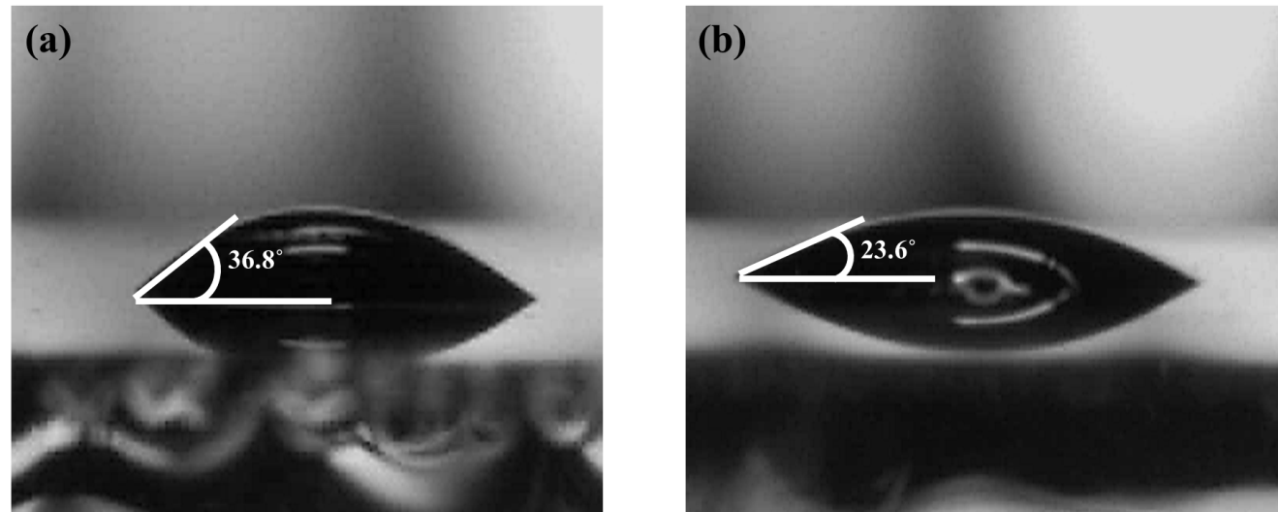


**Figure S1.** Contact angles of a water droplet on (a) PEDOT:PSS film and (b) ZnCo_2_O_4_ NPs layer deposited on FTO substrates.


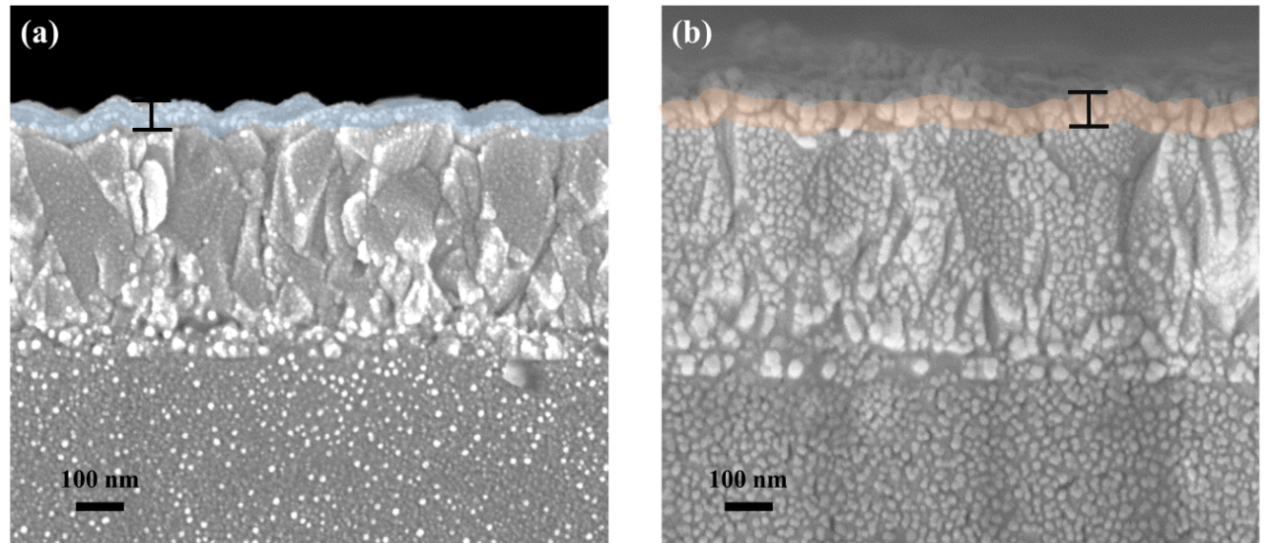


**Figure S2.** Cross-sectional SEM images of (a) PEDOT:PSS film and (b) ZnCo_2_O_4_ NPs layer deposited on FTO substrates.


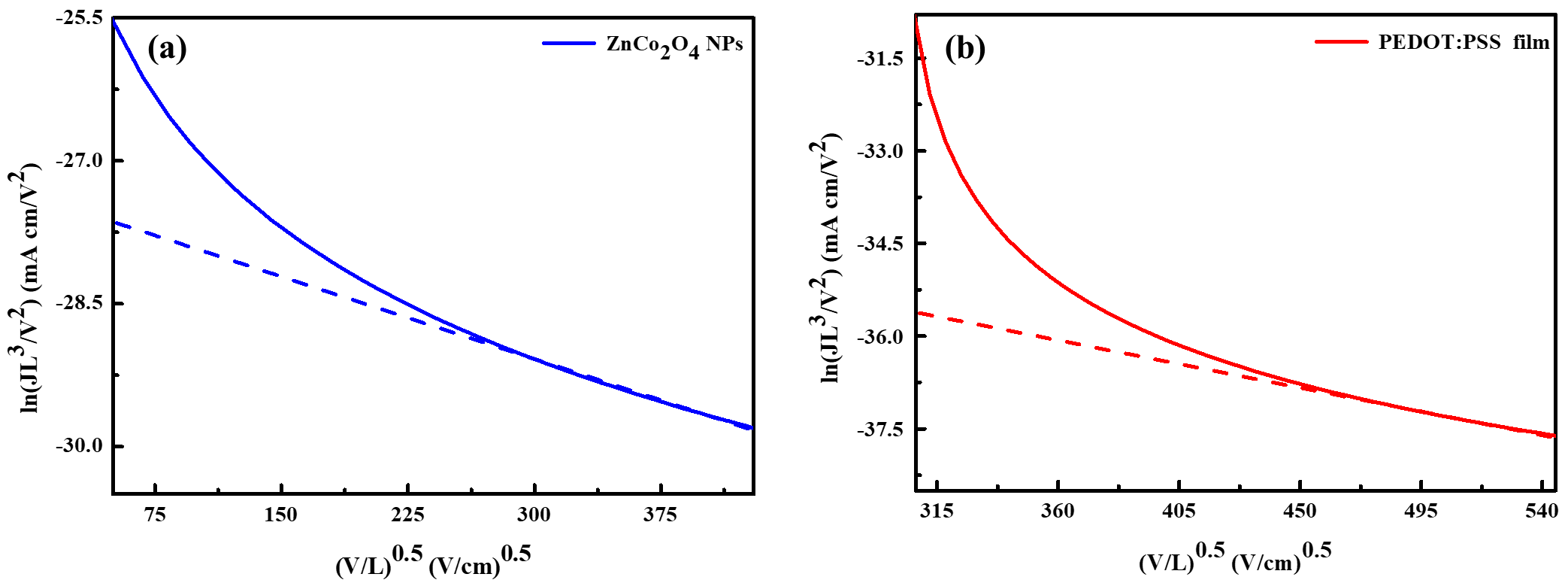


**Figure S3.** Hole mobility of (a) ZnCo_2_O_4_ NPs layer and (b) PEDOT:PSS film versus electric field (*V/L*)^0.5^.


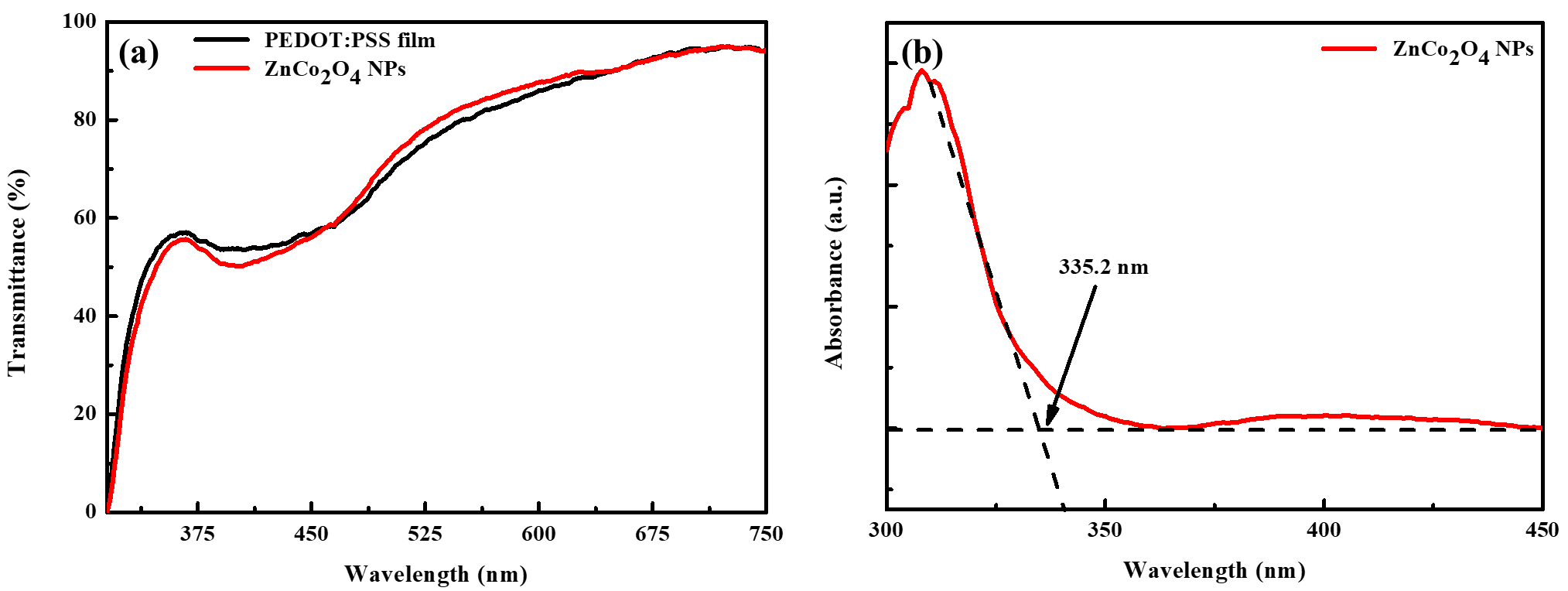


**Figure S4.** (a) Transmission spectra of PEDOT:PSS film and ZnCo_2_O_4_ NPs layer and (b) absorption spectra of ZnCo_2_O_4_ NPs layer.


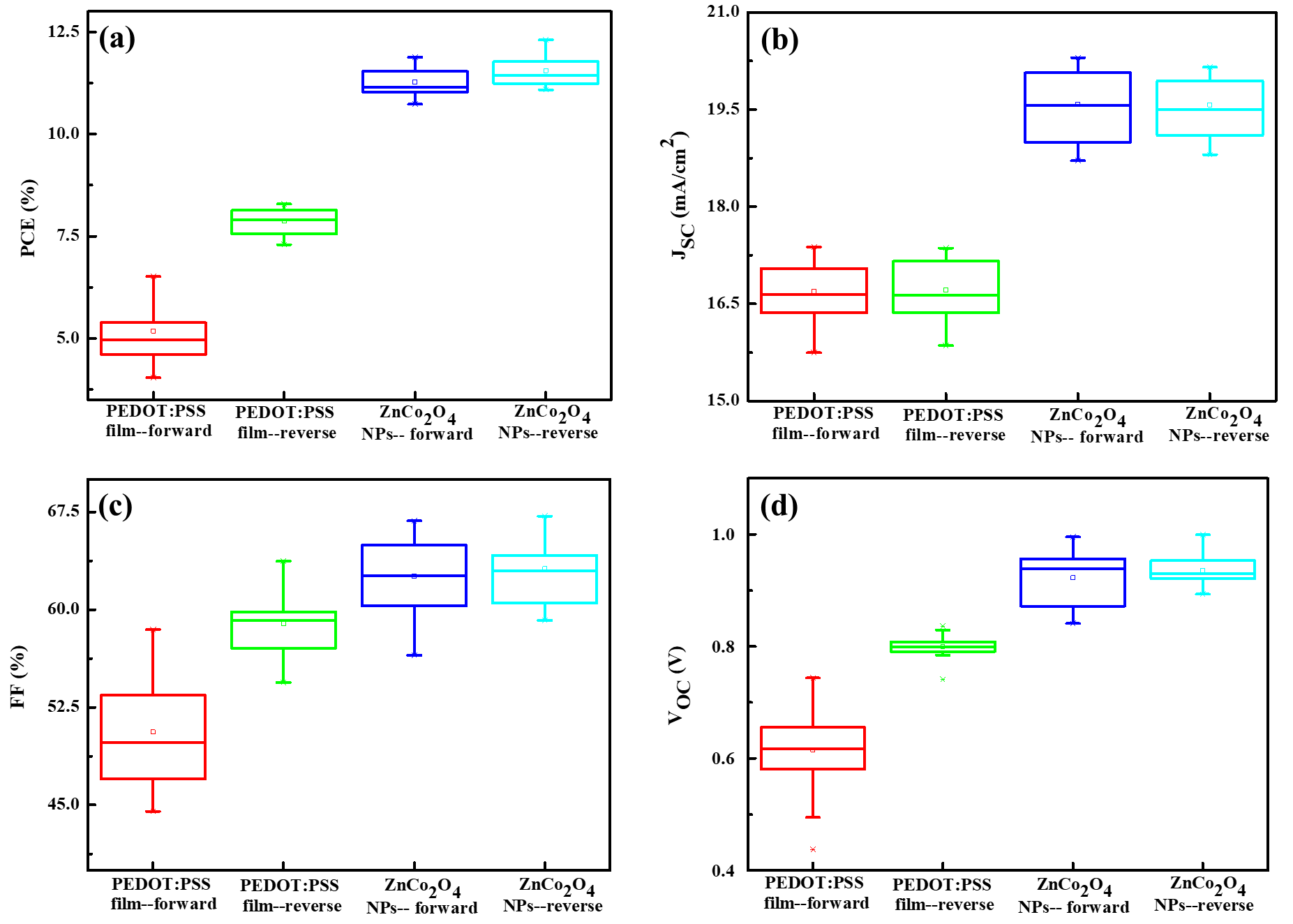


**Figure S5.** Performance variation represented as a standard box plot in (a) *PCE*, (b) *J*_SC_, (c) *FF*, and (d) *V*_OC_ from 20 devices based on PEDOT:PSS film and ZnCo_2_O_4_ NPs layer under forward and reverse scans.
